# A Report of Epidemics and Endemics—Cholera

**Published:** 1861-06

**Authors:** 


A Report on Epidemics and Endemics.—Cholera.—By O. C. Gibbs, M. D., Frewsburg, N. Y. 1861.

We are indebted to the author for a copy of this, the second paper in his proposed work on the various diseases ranked under the heads of Epidemics and Endemics, and which, first giving historical sketches of the rise, progress and theories of this class of diseases, was to have been followed by a full consideration of the modes of *treatment, etc.,*—the whole forming a systematic work on the subject.

This paper is marked by the author's usual broad, philosophical grasp of thought and treatment,—comprehensive and practical in scope and detail, and furnishing a strong appeal of facts and able argument alike to the popular and professional mind. Rejecting the theory that this voiceless and invisible plague, the death-spawn of an Indian jungle, is a neccessary part of the Divine economy—a theory worthy an African fetish or obi-worshipper, worthy the heathen estimate of the attributes of a heathen god,—Dr. Gibbs proceeds to show that it is the legitimate sequence of man's folly, arising from the vicissitudes of his physical and social conditions, preventable by his wisdom and amenable to common, universal sanitary laws. Upon which premises he bases the dicta, that judicious and efficient sanitary regulations and the free use of disinfectants, as prophylactic measures, will doubtless be of more service than any treatment; that the administration of chlorine would form a part of all judicious medication, citing the fact that Dr. Mann, of Clerkenwell, exhibited chlorine water in nearly one hundred cases, with a loss of only two patients.

We regret, exceedingly, the necessity which causes Dr. Gibbs to forego his original intention, and trust he may soon find himself at liberty to continue his valuable contributions to a branch of our literature, than which few others have been more neglected—*i. e.*, Epidemiology and its complement.

